# THE RELATIONSHIP BETWEEN THE FEAR OF FALLING AND QUALITY OF LIFE IN NURSING HOME RESIDENTS: THE ROLE OF ACTIVITY RESTRICTION

**DOI:** 10.1093/geroni/igac059.2570

**Published:** 2022-12-20

**Authors:** Dongjuan Xu, Yaqi Wang, Shanshan Zhu, Kefang Wang

**Affiliations:** Purdue University, West Lafayette, Indiana, United States; Shandong University, Jinan, Shandong, China (People's Republic); Shandong University, Jinan, Shandong, China (People's Republic); Shandong University, Jinan, Shandong, China (People's Republic)

## Abstract

Falls among older adults are a significant later life course event with detrimental impacts on health, quality of life (QOL), and mortality. Fear of falling (FOF) is a common problem, which can lead to physical and social activity restriction and poor QOL. However, few studies have comprehensively explored the relationships among FOF, activity restriction, and QOL among nursing home residents. This study aimed to investigate: 1) whether FOF is negatively associated with QOL; and 2) the mediating role of activity restriction in the relationship between FOF and QOL among nursing home residents in mainland China. This is a cross-sectional study. A total of 316 residents from 27 nursing homes participated in this study. The mixed-effects multivariate linear or ordinal logistic regression models were conducted. All statistical models were adjusted for resident- and facility-level variables. More than half of nursing home residents (55.4%) reported they often or always reduced their activities. As expected, FOF was directly associated with lower levels of QOL (β = -0.19, p = 0.010). However, the strength of this association was attenuated and no longer significant with the inclusion of activity restriction in the model. FOF was positively associated with activity restriction (β = 0.33, p < 0.001) and activity restriction was negatively associated with QOL (rarely/sometimes reduced activity: β = -1.23, p = 0.386; often/always reduced activity: β = -3.85, p = 0.014). Given the aging of our populations, it is important to take FOF and activity restriction into account when caring for older people.

